# Total psoriasis regression in a patient with chronic myeloid leukemia treated using nilotinib

**DOI:** 10.1007/s00277-025-06228-x

**Published:** 2025-02-28

**Authors:** Tomáš Horňák, Daniela Žáčková, Jiří Mayer

**Affiliations:** Department of Internal Medicine Hematology and Oncology, Faculty of Medicine, University Hospital Brno, Masaryk University, Brno, Czech Republic

**Keywords:** Chronic myeloid leukemia, Tyrosine kinase inhibitors, Nilotinib, Psoriasis, Adverse effects

## Abstract

Chronic myeloid leukemia (CML) treatment has been revolutionized over last 20 years because of tyrosine kinase inhibitors (TKIs) and patients with CML now have life expectancy of general population. Chronic TKI treatment may cause adverse effects (AEs) that vary in frequency and severity. Nilotinib shows common AEs with metabolic changes and skin AEs. Here, we present a case report of patient who experienced full psoriasis regression while treated with nilotinib. Dose reductions and an attempt to stop nilotinib treatment resulted in prompt psoriasis reappearance. This is the first documented case of positive effect of nilotinib on psoriasis.

## Introduction

Over the last 20 years, tyrosine kinase inhibitor (TKI) therapy has revolutionized cancer treatment; chronic myeloid leukemia (CML) is considered a well-controlled disease, with treatment-free remission (TFR) being the new proposed goal of CML therapy, [1] and overall survival of patients with CML is nearly similar to that of the general population. Unfortunately, only a few patients achieve stable TFR, and even in successful TFR cases, TKI withdrawal syndrome (TWS) may occur as a polymorphic set of symptoms associated with TKI cessation.[2] Therefore, most patients require life-long therapy, which causes adverse effects (AEs). Some AEs are common to all TKIs, whereas others show varied incidence rates, severity, and duration depending on the TKI administered. AEs such as arterial occlusion may be fatal in patients who receive nilotinib or ponatinib.[3] TKIs have an “off-target” effect on tyrosine kinases other than BCR::ABL1; however, the etiopathogenesis of AEs associated with TKIs remains unclear.[4] TKI dose reduction is proposed to minimize TKI-induced toxicity, which appears to be feasible based on data of patients with an optimal therapeutic response.[5].

Cutaneous AEs are common in patients who receive nilotinib. Three case reports in the literature have described onset of psoriasis during nilotinib treatment.[6] We describe a patient with a history of psoriasis, who was newly diagnosed with CML and observed disappearance of all psoriatic plaques during nilotinib treatment with recurrence of lesions after planned treatment discontinuation.

## Case report

**Fig. 1:**
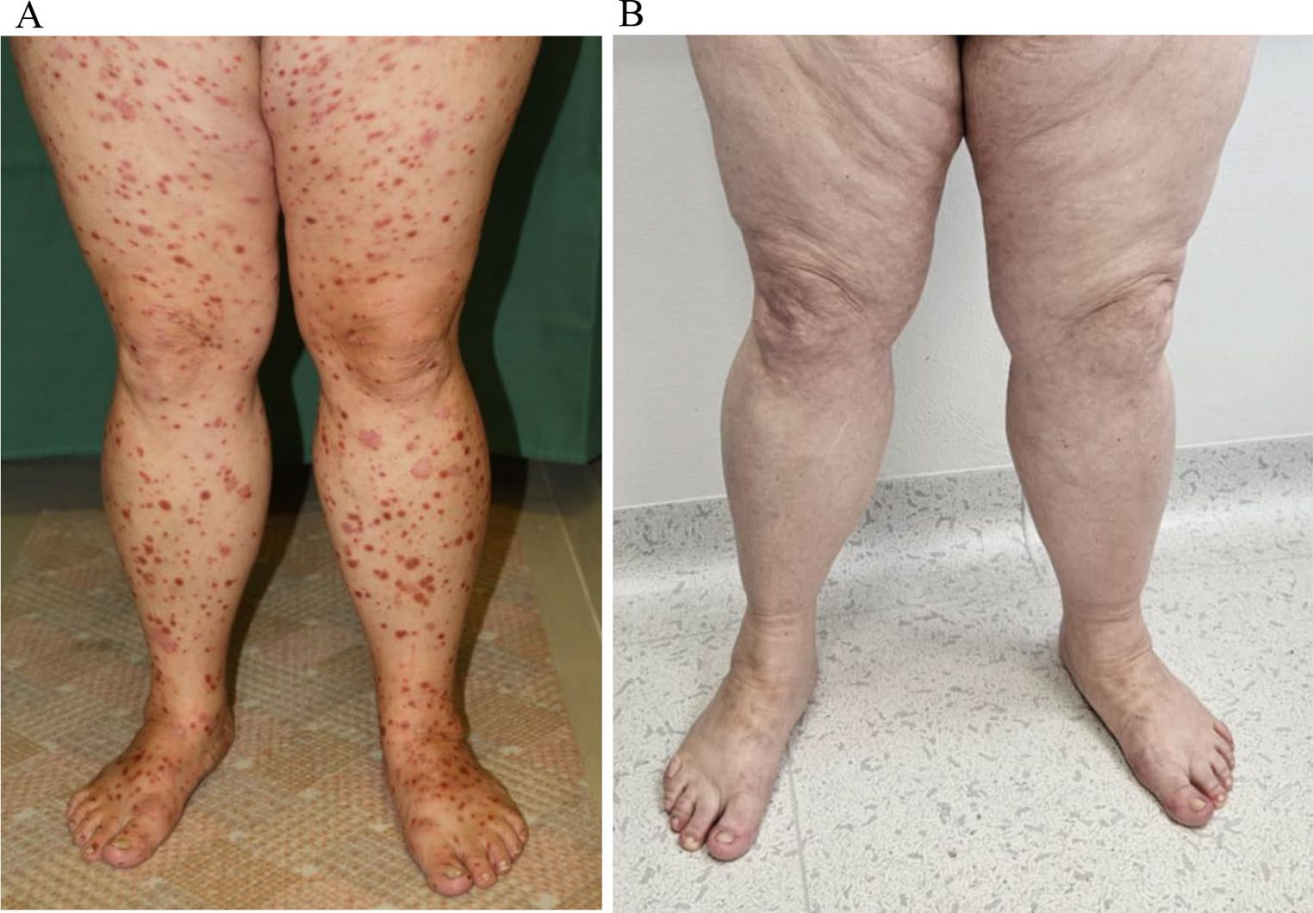
**A**- Patient taken photo at the time of psoriasis diagnosis, before CML treatment. **B**- Patient taken photo after nilotinib treatment

A woman born in 1959 was referred to our department in November 2012 for evaluation of newly diagnosed chronic-phase CML with an intermediate risk Sokal score. Her medical history included psoriasis diagnosed in 2011 with skin and joint involvement, benign thyroid nodules, and depression. She had histologically verified psoriatic lesions throughout her body at the time of CML diagnosis (Fig. 1A), and methotrexate and local corticosteroids were used as active treatment; she had received cyclosporine A and retinoids (both topical and systemic) previously.

Nilotinib (300 mg twice daily) was administered as first-line treatment after a short-term course of hydroxyurea and anagrelide cytoreductive therapy. Following nilotinib treatment initiation in December 2012, the patient observed rapid and complete regression of all psoriatic lesions over 1 month. Therefore, both methotrexate and topical treatment were discontinued, without recurrent lesions. The patient achieved a complete hematological, cytogenetic, and major molecular response after 1-, 4-, and 7-month therapy, respectively. Low-grade AEs (swelling, fatigue) necessitated adjustment of the nilotinib dose to 450 mg/day.

The patient was transported to the emergency room for evaluation of new-onset chest pain in October 2013. She was diagnosed with hypertension and received treatment and had no signs of cardiac ischemia. She achieved a deep molecular response (DMR) during this period. The patient was diagnosed with diabetes, 3 months later (1 year after nilotinib initiation). Considering a probable association with nilotinib therapy, we suggested switching treatment to an alternative TKI; however, the patient preferred to continue nilotinib therapy, and we reduced the dose to 300 mg/day. Psoriatic lesions recurred a week after dose reduction, necessitating dose re-adjustment to 450 mg daily, which led to immediate regression of lesions (Fig. 1B). The patient developed acute myocardial infarction, 14 months following nilotinib therapy initiation. Coronaroplasty and stent insertion were performed, and dual anti-aggregation therapy was prescribed. We advised switching treatment to imatinib; however, she refused based on her fear of psoriasis recurrence. Re-attempted reduction of the nilotinib dose to 300 mg daily was successful, and the patient continued nilotinib treatment for over 6 years without any new AEs; the DMR was retained, and she had an overall good clinical response.

**Fig. 2:**
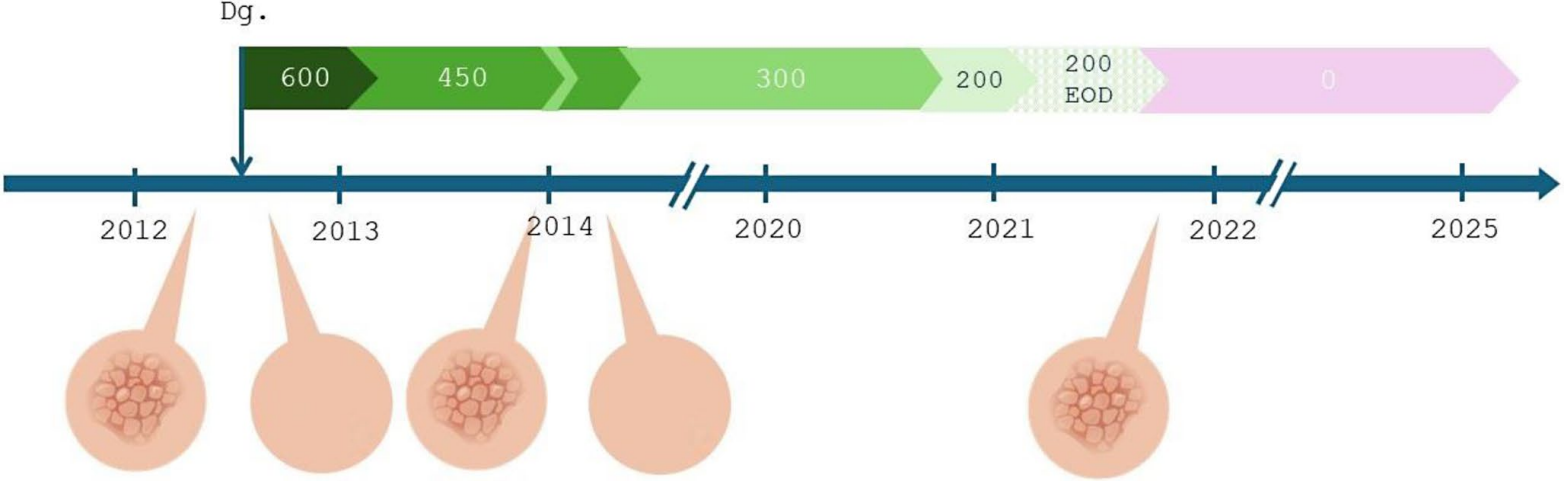
Scheme demonstrating the association between nilotonib treatment/ dosage (mg) and psoriatic lesions. Dg- diagnosis, EOD- every other day

The patient was enrolled in the nationwide prospective investigator-initiated phase II clinical trial HALF (ClinicalTrials.gov NCT04147533) in 2020, with the aim to investigate the efficacy and safety of TKI discontinuation after previous two-step dose reduction in patients with CML with DMR. The first dose reduction to 200 mg of nilotinib daily for 6 months was followed by 200 mg administered every other day for the subsequent 6 months during the second step. Both dose reductions were well tolerated, without recurrent psoriatic lesions or TKI withdrawal syndrome, and the DMR was retained. However, the patient quickly developed generalized recurrence of psoriatic skin lesions after complete discontinuation of nilotinib therapy and was admitted for inpatient treatment. Methotrexate therapy showed only a partial effect. During the TFR phase, the patient was diagnosed with bronchogenic carcinoma, which was completely resected surgically, followed by involved-field irradiation therapy. Currently, the patient has been in a stable TFR since cessation of nilotinib treatment in August 2021 and has done well overall, with only mild psoriatic findings (Fig. 2).

## Discussion and summary

Despite successful treatment of CML and achievement of stable TFR in our patient, this case report highlights the challenges associated with modern CML therapy. Currently, six TKIs are available worldwide, each with a different spectrum of AEs. The off-target effects of TKIs on other tyrosine kinases may contribute to AEs.[4] Even non-fatal AEs can significantly reduce patients’ quality of life and therapy compliance.

Three case reports in the literature have described an association between nilotinib treatment and psoriasis. Some authors have proposed that nilotinib inhibits regulatory T-lymphocytes; however, this theory remains unsubstantiated.[6] Our case report is the first to document total regression of psoriasis using nilotinib therapy, which is supported by the fact that complete withdrawal of the drug led to recurrent lesions. This can be considered a unique symptom of TWS. We speculate that other tyrosine kinases inhibited by nilotinib may be involved in cell signalling and development of psoriasis.

However, the pathophysiology of psoriasis remains unclear. Cytokine and T-cell driven responses contribute to perpetuation of autoimmunity. The tumor necrosis factor α–interleukin 23–Th17 axis plays a central role in most cases of common plaque psoriasis, whereas other variants (guttate, pustular, or nail psoriasis) show diverse contributory pathophysiological mechanisms. Discoidin domain receptor 1 (DDR1) promotes migration of Th17 cells. The DDR1 receptor tyrosine kinase is strongly inhibited by nilotinib, which may be a mechanism underlying psoriasis regression.[7].

CML therapy is subject to a patient-specific design. Treatment response and toxicity are common variables that affect the choice and dosage of TKIs. Patient preference also plays an important role. Throughout the course of therapy, our patient was advised to switch treatment to an alternative TKI (imatinib) considering the risk of AEs. However, she preferred nilotinib treatment to avoid the risk of psoriasis relapse. The patient changed her opinion years later when she was offered the choice of participation in a TKI cessation trial.

Although not generally recommended, long-term TKI dose reduction, as attempted in our patient, has been proposed to minimize AE incidence while maintaining the treatment response and may be a long-term aim in patients who are unsuitable for or unable to achieve TFR.[5] Further studies are warranted to investigate this possibility; we intend to report feasibility of this strategy with results from the HALF study.

In summary, we present the first documented case of nilotinib-induced complete regression of psoriasis in a patient with CML. We conclude that TKI dose reduction may be feasible in many patients with CML to reduce the risk of AEs and that the patient’s decision should be an important component of the therapeutic process.

## Data Availability

No datasets were generated or analysed during the current study.
